# The Role of Lung Ultrasound in Diagnosing COVID-19-Related Multisystemic Inflammatory Disease: A Preliminary Experience

**DOI:** 10.3390/jcm11010234

**Published:** 2022-01-01

**Authors:** Anna Maria Musolino, Elena Boccuzzi, Danilo Buonsenso, Maria Chiara Supino, Maria Alessia Mesturino, Eugenio Pitaro, Valentina Ferro, Raffaella Nacca, Serena Sinibaldi, Paolo Palma, Alberto Villani, Paolo Tomà

**Affiliations:** 1 Pediatric Emergency, Department of Emergency and General Pediatrics, Bambino Gesù Children’s Hospital, IRCCS, 00100 Rome, Italy; amcaterina.musolino@opbg.net (A.M.M.); elena.boccuzzi@opbg.net (E.B.); mariachiara.supino@opbg.net (M.C.S.); malessia.mesturino@opbg.net (M.A.M.); valentina.ferro@opbg.net (V.F.); raffaella.nacca@opbg.net (R.N.); 2 Department of Woman and Child Health and Public Health, Fondazione Policlinico Universitario A. Gemelli IRCCS, 00100 Rome, Italy; 3 Global Health Research Institute, Università Cattolica del Sacro Cuore, 00100 Roma, Italy; 4 Academic Department of Pediatrics, Bambino Gesù Children’s Hospital, IRCCS, 00100 Rome, Italy; pitaroeugenio@gmail.com; 5 Pediatric Unit, Department of Emergency and General Pediatrics, Bambino Gesù Children’s Hospital, IRCCS, 00050 Palidoro, Italy; serena.sinibaldi@opbg.net; 6 Clinical Immunology and Vaccinology Unit, Research Unit in Clinical Immunology and Vaccinology, Academic Department of Pediatrics, Bambino Gesù Children’s Hospital, IRCCS, 00100 Rome, Italy; paolo.palma@opbg.net; 7 Department of Emergency and General Pediatrics, Bambino Gesù Children’s Hospital, IRCCS, 00100 Rome, Italy; alberto.villani@opbg.net; 8 Department of Imaging, Bambino Gesù Children’s Hospital, IRCCS, 00100 Rome, Italy; paolo.toma@opbg.net

**Keywords:** lung ultrasound, multisystem inflammatory syndrome, children

## Abstract

Background: To date, there are no data regarding the systematic application of Point-of-Care Lung Ultrasound (PoC-LUS) in children with Multisystem Inflammatory Syndrome in Children (MIS-C). The main aim of this study is to show the role of Point-of-Care Lung Ultrasound as an additional aid in the diagnosis of COVID-19-related Multisystem Inflammatory Syndrome in Children (MIS-C). Methods: Between April 2020 and April 2021, patients aged 0–18 years referred to our emergency department for fever, and later hospitalized without a specific diagnosis, underwent PoC-LUS. Ultrasound images of patients with a final diagnosis of MIS-C were retrospectively evaluated. Results: Ten patients were enrolled. All were described to have pleural irregularities and B-lines. In particular: 8/10 children presented with isolated B-lines in at least half of the lung areas of interest; 8/10 presented with multiple B-lines and 3/8 had them in at least 50% of lung areas; 5/10 had a white lung appearance in at least one lung area and 1/5 had them in half of the areas of interest. Pleural effusion was described in 9/10. Conclusions: During the ongoing COVID-19 pandemic, we suggest performing PoC-LUS in febrile patients with high levels of inflammatory indices and clinical suspicion of MIS-C, or without a certain diagnosis; the finding of many B-lines and pleural effusion would support the diagnosis of a systemic inflammatory disease.

## 1. Introduction

Point-of-Care Lung Ultrasound (PoC-LUS) is an increasingly applied tool to be used in the overall assessment of patients in pediatric Emergency Departments (ED). Thanks to its safety profile, simplicity of application (after adequate training), its high sensitivity and specificity, and by limiting exposure to ionizing radiation, lung ultrasound (LUS) may be used at the bedside for detecting many pathological findings. 

These features, together with the lack of need to transfer the patient to a traditional imaging environment, have allowed LUS to further extend its application, especially in response to the restrictive measures in place due to COVID-19. Indeed, new roles for PoC-LUS have been defined for triaging confirmed COVID-19 pediatric patients, detecting pneumonia in the context of SARS-CoV-2 pulmonary involvement, monitoring the evolution of lung impairment, and for the effectiveness of therapies [1,2].

It is widely known that children represent only a small number of COVID-19 cases worldwide, and that they are usually asymptomatic or show milder symptoms than adults [3,4]. However, concurrent with the outbreak of COVID-19 infections, since mid-late April 2020 a severe multisystemic inflammatory condition leading to multiorgan failure and shock, and temporally associated with SARS-CoV2 infection, began to be increasingly described in Europe and North America. The new clinical entity was later named Pediatric Inflammatory Multisystem Syndrome: temporally associated with SARS-CoV-2 (PIMS-TS) by the Royal College of Pediatrics and Child Health (RCPCH) [5] and Multisystem Inflammatory Syndrome in Children (MIS-C) by the United States Centers for Disease Control (CDC) [6] and World Health Organization (WHO) [7].

Several papers and reviews on clinical features, laboratory evaluation, imaging findings, therapeutic management, clinical course and outcome in MIS-C have been published over the months. However, due to the primarily extrapulmonary symptoms of SARS-CoV-2-infection, there are few works that focus on analysis of lung imaging findings. Moreover, all the published works refer to traditional tools, i.e., chest X-ray (CXR) and chest Computed-Tomography (CT) [8,9,10]. Only Kennedy et al., as far as we know, have currently reported the PoC-LUS findings in MIS-C [11]. Others have only speculated on its application based on the experiences of COVID-19-patients [12].

We report our experience to support this new idea of applying PoC-LUS in the diagnostic path of MIS-C. 

## 2. Materials and Methods

An observational study was conducted at the ED of Bambino Gesù Children’s Hospital over a period of one year (April 2020–April 2021). The study was approved by the Ethics Committee of Bambino Gesù Children Hospital (2111_OPBG_2020). Patients aged 0–18 years admitted to ED for fever, and subsequently hospitalized without a specific diagnosis, underwent PoC-LUS as an integrative diagnostic aid to clinical, laboratorial and instrumental evaluation. For our study, we retrospectively evaluated the ultrasound (US) images of all patients who underwent PoC-LUS in the ED and were later diagnosed with MIS-C after admission.

The finding of respiratory or systemic infections was considered an exclusion criterion.

PoC-LUS was performed by only five Pediatric Emergency Medicine (PEM) physicians, with more than 5 years of Point-of-Care Ultrasound (PoCUS) experience and equipped with personal protective equipment. 

An ultrasound pocket device (Sonosite iViz; FUJIFILM Sonosite, Amsterdam, The Netherlands) with a 10–5 MHz linear probe covered with single-use transparent plastic disposable material and ultrasound transmission gel in single-use package were used for examinations, followed by adequate sterilization procedures [13].

Patients underwent US evaluation in a sitting position and 10 areas (2 anterior, 2 posterior and 1 axillar area for each hemithorax) were scanned. The US findings considered were: pleural effusion, pleural irregularities (including sub-pleural consolidations), parenchymal consolidations and B-lines; B-line density was defined by the finding of multiple B-lines, while white lung was defined as increased lung echogenicity with disappearance of normal A-lines. 

A descriptive analysis including the absolute number and percentage for qualitative variables, plus calculation of central tendency (mean) and dispersion (standard deviation) was conducted.

## 3. Results

Ten patients evaluated with PoC-LUS in the ED were later diagnosed to have MIS-C after admission. 

The mean age was 9.01 ± 1.24 years and 5/10 (50%) were male. All presented with fever lasting 5.9 ± 0.77 days and 8/10 (80%) had been on antibiotic therapy for at least 48 h (Table 1). Gastrointestinal disorders were the most frequent symptom (70%): abdominal pain was present in five children (50%), vomiting in three cases (30%) and diarrhea in the same percentage (30%); analyzing the associations between the above symptoms, two patients presented with both abdominal pain and vomiting, two with pain and diarrhea, one with pain, one with vomiting and one with diarrhea only (Table 1). A total of 40% of the children (4/10) had skin manifestations. Musculoskeletal symptoms were reported in 40% (4/10): arthralgia and myalgia were present in three and two patients, respectively, and one child presented with both. Respiratory symptoms were also present in 40% (4/10): cough and dyspnea were reported in 30% and 20% of the patients, respectively, and one child presented with both. Finally, 20% of the patients had headaches, and the same percentage presented with conjunctivitis (Table 1). Most of the patients (7/10) reported a link to SARS-CoV-2: four patients had a history of contact with a known case of COVID-19, and three reported having had the infection, with a minimum and maximum interval of symptom presentation of 12 and 60 days, respectively.

Within 12 h of clinical evaluation, all patients underwent complete blood count, C-reactive protein (CRP), ferritin, fibrinogen and International Normalized Ratio (INR), while brain natriuretic peptide (BNP) and troponin were performed in 9/10, and erythrocyte sedimentation rate (ESR) in 4/10 (40%). The mean value of white cell counts (WCC) was 8880 ± 1086/μL; granulocyte and lymphocytes counts were 7023 ± 996/μL and 1057 ± 112/μL, respectively (Table 2). CRP was increased with a mean value of 11.44 ± 1.8 mg/dL; ferritin and fibrinogen were also higher than normal: 2958 ± 2549 ng/dL and 594 ± 48.4 mg/dL, respectively (Table 2). The mean value of BNP was 1770 ±533 pg/mL, and that of troponin was 46 ±16 pg/mL (Table 2). The mean values of other indices (e.g., ESR, D-dimers, procalcitonin) were not calculated because they were performed in only a small percentage of patients. All patients had serological evidence of a previous SARS-CoV-2 infection.

**Figure 1:**
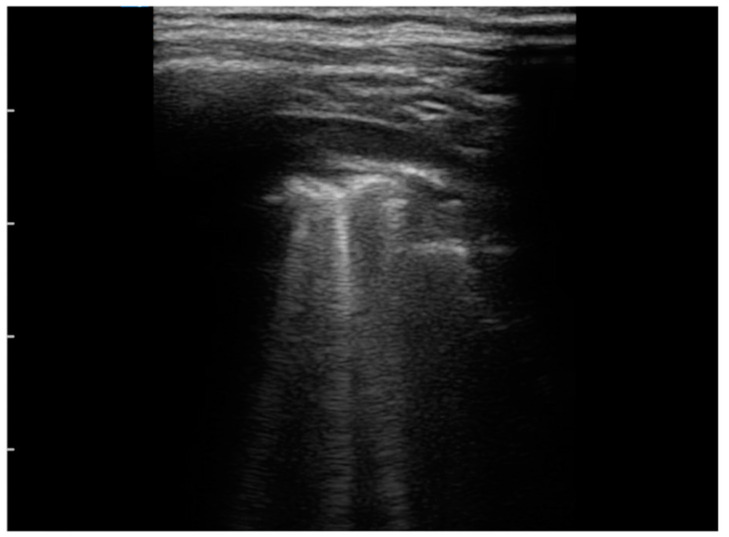
Pleural effusion < 1 cm, pleural irregularity and several B-lines.

**Figure 2:**
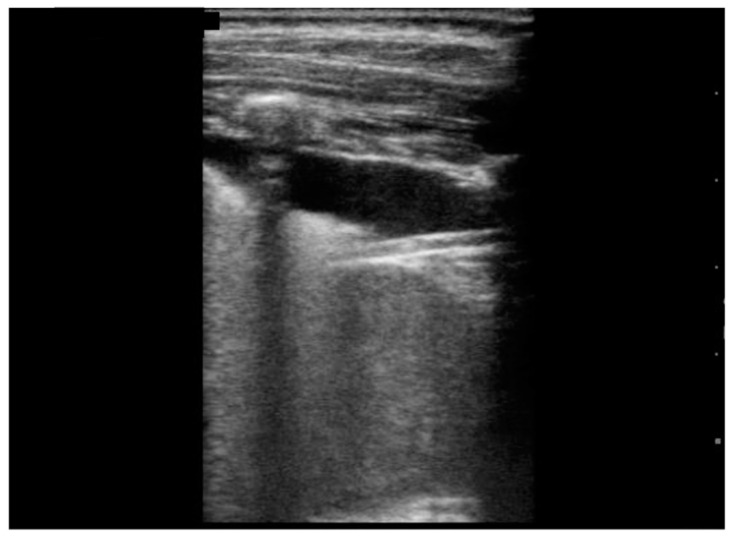
Pleural effusion < 1 cm, white lung.

**Figure 3:**
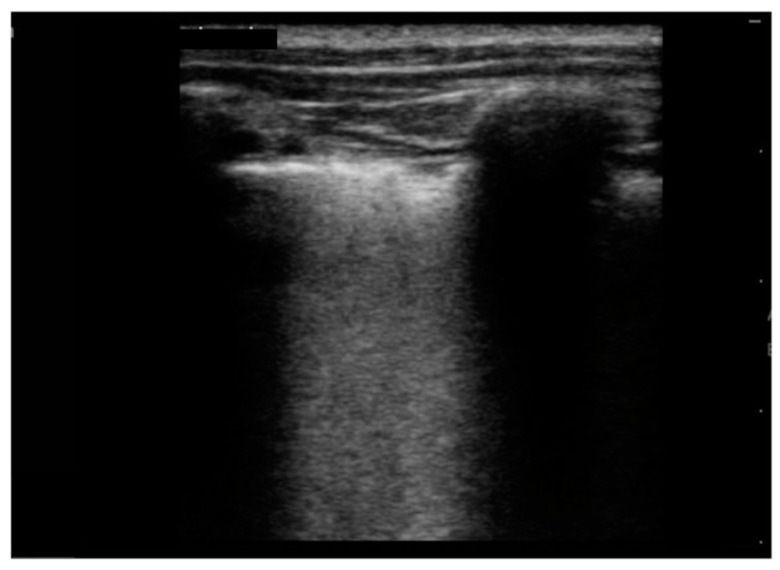
Subpleural consolidation < 1 cm, white lung.

As for the US findings (Table 3), all patients were described to have pleural irregularities (Figure 1) and isolated B-lines. In particular, 8/10 children presented with isolated B-lines in at least half of the lung areas of interest, and two of them had all ten areas involved; the minimum number of affected areas of interest was three described in one patient. The most frequent localization of B-lines was the left posterior basal and the left axillar areas (10 /10 patients), followed by the right posterior basal area (9/10). Moreover, 8/10 children presented with multiple B-lines (Figure 1) with three patients (37.5%) having such findings in at least 50% of lung areas. In 5/10 patients there was a white lung (Figure 2 and Figure 3) appearance in at least one lung area; in particular, 20% (1/5) had a white lung appearance in 5/10 areas of interest. Pleural effusion was described in 9/10 (90%), and was unilateral in 8/9 (89%) and bilateral in one child. Sub-pleural consolidations (Figure 3) were detected in 7/10 (70%) but turned out to be a minor US finding: in most cases (4/7; 57.1%) they were present in only one lung area (Table 3).

## 4. Discussion

Pediatric cases are reported as 2.1–7.8% of total COVID-19 cases [6], but the number may be underestimated as most children remain asymptomatic or have mild symptoms, and are tested less frequently than adults [14,15]. This is the main reason why the true incidence of MIS-C is unclear [15]. Due to the high proportion of patients with serological evidence of a previous SARS-CoV-2 infection, and its onset after 2–6 weeks [14,15] from COVID-19 infection, pathogenesis of MIS-C is thought to be a postinfectious immune-mediated host response. Thus, the understanding of the immune system’s involvement has warranted extensive study [16,17]. The pathogenetic mechanism leading to pathological pulmonary outcomes in MIS-C should be multifactorial, and mainly related to the depression of myocardial function associated with volume overload and to the enormous hyperinflammatory cytokine storm [10,18]. 

The definition of MIS-C presented by the WHO, CDC and RCPCH always consider the presence of fever, laboratory evidence of inflammation and multisystem involvement, but certain differences in criteria—including the requirement of documented infection—are inconsistent between the organizations [5,6,7]. 

Numerous articles and reviews have been published reporting mainly clinical and laboratory findings of the disease. According to these studies, the mean age of children with MIS-C ranges from 7 to 10 years [19,20,21,22,23,24]; we confirmed this data with the mean age of our patients (9.01 ± 1.24). The most common symptom is fever, which was reported in almost all patients [14,20,21,22,24,25,26,27], followed in frequency by gastrointestinal (GI) manifestations (abdominal pain, diarrhea and vomiting), which was detected in 70–88% [14,15,19,22,23,24,27]. Respiratory signs are generally not part of the MIS-C presentation, and are therefore found in a smaller percentage of children (4–55%) [14,15,19,22,23,24,26,27]. In our cases, all patients presented with fever and 70% of them had GI manifestations: mainly abdominal pain, which was reported in 5/7 children having GI symptoms. Only 4/10 had respiratory presentation, with the most common symptom being coughing, present in 3/4 who had respiratory presentation. In addition to the GI symptoms, cardiovascular indications are another peculiar finding in MIS-C: ranging from tachycardia to hypotension and shock [14,15,19,20,28]; in fact, markers of myocardial damage, such as troponins and BNP, are often elevated [15,19,20,21,22,23,24,27]. This evidence is confirmed by our data, which show values significantly higher than normal, especially for BNP. WCC is frequently increased [22,27], and is commonly associated with lymphocytopenia [12,15,20,21,22,23,24,27], but neutrophilia has also been observed [12,15,23,27]. Our data confirm the low lymphocyte count as the most significant pathological datum of the complete blood count. General inflammatory markers including procalcitonin [12,21,22,24,27], ESR [12,22,24,27], CRP and ferritin [12,15,19,20,21,22,24,27], are frequently upregulated, as are coagulation markers, including D-dimers and fibrinogen [12,15,19,20,22,24]. In particular, disease severity appears to be associated with different marker values, and therefore patients with severe MIS-C show increased levels of WBC, CRP, D-dimer and ferritin compared to patients with non-severe disease [29]. In our patients, CRP, ferritin and fibrinogen were also all increased above the normal values. Among our cases, seven children (70%) reported a link with SARS-CoV-2, and all had serological evidence of a previous COVID-19 infection with no co-infections. Of note, MIS-C has some overlapping clinical and laboratory features with Kawasaki Disease [16,30], and although some immunological differences have been highlighted, the differential diagnosis can remain difficult. For this reason, we cannot exclude the possibility that lung ultrasound may also detect ultrasound patterns of interstitial inflammation due to vascular overload in Kawasaki Disease also, as has recently been described by Buonsenso et al. in a study assessing cardiopulmonary interactions in children with systemic inflammatory diseases [31].

Less information regarding MIS-C imaging is available in the literature. The broader scope is obviously the cardiac system, as it is considered the most severe localization of the disease [32], but an important field of application is also gastrointestinal imaging, due to the rate of such localization. Irregular symmetrical infiltrates and pleural effusions are among the elements of the RCPCH case definition of MIS-C [5], but due to less frequent and usually not severe respiratory symptoms, only a few studies focus on radiological pulmonary findings. Indeed, pulmonary involvement in MIS-C is generally mild [12] and therefore CXRs are normal in about half of patients [10,23,30]. In pathological CXRs and CTs, diffuse pulmonary opacities/infiltrates [8,9,15,18,22]—both consolidations and ground glass opacities in severe cases [32]—and peribronchial thickening on CXR [8,18,33] are reported despite patients having few or no lower respiratory symptoms. Pleural effusion is widely described [9,18,24,33], and, together with a predominance of thickening in the lower lung area, is more characteristic in children with MIS-C than in children with COVID-19 [10,18]. Bilateral peripheral and subpleural opacities are usually present in children who have predominantly pulmonary disease during SARS-CoV-2 infection [8,18]. These significant and predominant manifestations may help simplify the diagnostic orientation between these two entities in the COVID-19 era. However, in view of the limited clinical relevance of pulmonary involvement in MIS-C, we believe it would be unethical to expose pediatric patients to ionizing radiation in the case of mild or absent respiratory symptoms. 

To the best of our knowledge, only one article reports data on lung PoCUS performed on pediatric MIS-C patients [11]. It describes US pathological images in 7/9 patients with MIS-C, and reports pleural effusion as the most frequent pathological finding. All our patients had pathological findings on LUS (Table 3), with the most significant US findings in MIS-C patients being B-lines in most lung fields and pleural effusion. In the COVID-19-era, this latter finding, in particular, may help PEM physicians differentiate between an ongoing infection and a systemic inflammatory disease, given the absence of effusion in the SARS-CoV-2 disease. However, it is worth emphasizing that the use of PoCUS in these patients requires a team of practitioners experienced in the methodology, and with appropriate certifications, plus the implementation of a local reporting system, ideally approved by the relevant national societies.

Our study presents clear limitations. The number of patients is low because not all children managed in our hospital for MIS-C underwent PoC-LUS in the ED due to the fact that LUS was not performed by all PEM physicians, but only by those who have had adequate training and felt comfortable performing PoCUS. This could lead to bias in the results. Furthermore, the number of cases did not allow us to make a statistical correlation between US findings and other data (e.g., blood tests related to evaluation of inflammatory status or cardiac compromise). Finally, a comparison between our US images and a control group is lacking.

## 5. Conclusions

In conclusion, based on our experience and data of literature [11], we are confident of the potential role of LUS for MIS-C patients. As previously suggested [12], during the ongoing COVID-19 pandemic we propose to perform PoC-LUS in all febrile patients with high levels of inflammatory indices and a clinical suspicion of MIS-C, or in the absence of a diagnostic orientation at ED. Indeed, the finding of bilateral diffuse B-lines associated with pleural irregularities and pleural effusions may suggest MIS-C, or may help guide diagnosis even in the absence of respiratory symptoms. 

Furthermore, performing PoC-LUS would avoid exposure to radiation, which—unethical in the absence of respiratory symptoms—has been restricted to children with respiratory distress. Finally, it is desirable to use the PoC-LUS during the course of the disease to monitor the state of pulmonary inflammation, whether or not the inflammation is solely an expression of systemic inflammation or if it is secondary to cardiac involvement. 

Certainly, US findings are not specific and need always to be related to other clinical and laboratory data. However, for the reasons described above, PoC-LUS may be an additional useful tool for PEM physicians. Further studies on LUS of pediatric MIS-C patients are needed to clarify the distinctive US features and to correlate them with clinical and laboratory data.

## Figures and Tables

**Table 1:** Clinical characteristics at presentation.

Age (years), mean ± SD	9.01 ± 1.24
Sex, *n* (%)	
Female	5 (50)
Male	5 (50)
Duration of fever (days), at admission, mean ±SD	5.9 ± 0.77
On antibiotic treatment, *n* (%)	8 (80)
History of contact with COVID case, *n* (%)	4 (40)
History of COVID infection, *n* (%)	0
Gastrointestinal symptoms (tot), *n* (%)	7 (80)
Abdominal pain, *n* (%)	5 (50)
Diarrhea, *n* (%)	3 (30)
Vomit, *n* (%)	3 (30)
Respiratory symptoms (tot), *n* (%)	4 (40)
Cough, *n* (%)	3 (30)
Dyspnea, *n* (%)	2 (20)
Sore throat, *n* (%)	0
Skin signs, *n* (%)	4 (40)
Musculoskeletal symptoms (tot), *n* (%)	4 (40)
Arthralgia, *n* (%)	3 (30)
Myalgia, *n* (%)	2 (20)
Conjunctivitis, *n* (%)	2 (20)
Headache, *n* (%)	2 (20)
Thorax pain, *n* (%)	0

**Table 2:** Laboratory findings.

	Mean ± SD
White blood cell (/µL)	8880 ± 1086
Granulocyte count (/μL)	7023 ± 996
Lymphocytes (/µL)	1057 ± 112
CRP (mg/dL)	11.44 ± 1.8
Ferritin (ng/dL)	2958 ± 2549
Fibrinogen (mg/dL)	594 ± 48.4
BNP (pg/mL)	1770 ± 533
Troponin (pg/mL)	46 ±16
INR	1.11 ± 0.1

**Table 3:** LUS findings.

	N. Patients (Tot 10; %)	N. Lung Areas (Tot 10; %)
Irregular/indented/broken pleural line, *n* (%)	10 (100)	
B-lines, *n* (%)	10 (100)	
B-lines	2 (20)	10 (100)
B-lines	2 (20)	8 (80)
B-lines	1 (10)	6 (60)
B-lines	3 (30)	5 (50)
B-lines	1 (10)	4 (40)
B-lines	1 (10)	3 (30)
Multiple/several B-lines, *n* (%)	8 (80)	
White lung, *n* (%)	5 (50)	
Sub-pleural consolidation, *n* (%)	7 (70)	
Sub-pleural consolidation	4 (57.1)	1 (10)
Sub-pleural consolidation	2 (28.6)	2 (20)
Sub-pleural consolidation	1 (14.3)	4 (40)
Pleural effusion, *n* (%)	9 (90)	
LUS score, mean ± SD	10.5± 1.81	

## Data Availability

Available upon request.
